# A non-parametric Bayesian model for joint cell clustering and cluster matching: identification of anomalous sample phenotypes with random effects

**DOI:** 10.1186/1471-2105-15-314

**Published:** 2014-09-24

**Authors:** Murat Dundar, Ferit Akova, Halid Z Yerebakan, Bartek Rajwa

**Affiliations:** Computer Science Department, IUPUI, 723 W. Michigan St, 46037 Indianapolis, IN US; Bindley Bioscience Center, Purdue University, 1203 W. State Street, 47907 W. Lafayette, IN US

**Keywords:** Anomaly detection, Sample characterization, Clustering, Cluster matching, Random effects, Meta clusters, Non-parametric Bayesian

## Abstract

**Background:**

Flow cytometry (FC)-based computer-aided diagnostics is an emerging technique utilizing modern multiparametric cytometry systems.

The major difficulty in using machine-learning approaches for classification of FC data arises from limited access to a wide variety of anomalous samples for training. In consequence, any learning with an abundance of normal cases and a limited set of specific anomalous cases is biased towards the types of anomalies represented in the training set. Such models do not accurately identify anomalies, whether previously known or unknown, that may exist in future samples tested. Although one-class classifiers trained using only normal cases would avoid such a bias, robust sample characterization is critical for a generalizable model. Owing to sample heterogeneity and instrumental variability, arbitrary characterization of samples usually introduces feature noise that may lead to poor predictive performance. Herein, we present a non-parametric Bayesian algorithm called ASPIRE (*a*nomalous *s*ample *p*henotype *i*dentification with *r*andom *e*ffects) that identifies phenotypic differences across a batch of samples in the presence of random effects. Our approach involves simultaneous clustering of cellular measurements in individual samples and matching of discovered clusters across all samples in order to recover global clusters using probabilistic sampling techniques in a systematic way.

**Results:**

We demonstrate the performance of the proposed method in identifying anomalous samples in two different FC data sets, one of which represents a set of samples including acute myeloid leukemia (AML) cases, and the other a generic 5-parameter peripheral-blood immunophenotyping. Results are evaluated in terms of the area under the receiver operating characteristics curve (AUC). ASPIRE achieved AUCs of 0.99 and 1.0 on the AML and generic blood immunophenotyping data sets, respectively.

**Conclusions:**

These results demonstrate that anomalous samples can be identified by ASPIRE with almost perfect accuracy without *a priori* access to samples of anomalous subtypes in the training set. The ASPIRE approach is unique in its ability to form generalizations regarding normal and anomalous states given only very weak assumptions regarding sample characteristics and origin. Thus, ASPIRE could become highly instrumental in providing unique insights about observed biological phenomena in the absence of full information about the investigated samples.

**Electronic supplementary material:**

The online version of this article (doi:10.1186/1471-2105-15-314) contains supplementary material, which is available to authorized users.

## Background

### Automated analysis of cytometry data

Flow cytometry (FC) is a leading technology for cell analysis, allowing rapid evaluation of heterogeneous cellular populations in a single-cell setting, i.e., interrogating separately every individual cell in a sample. The analysis process uses fluorescently labeled antibodies to tag cellular epitopes known from their association with a specific cell function or state. This methodology in combination with various probes for cell viability, structure, and function can provide information-rich data sets describing the phenotypic effects of various natural physiological phenomena or the impact of external perturbants on characteristics of cell populations [1]. FC-based single-cell analysis is employed in various fields of life sciences ranging from immunology, hematology, and oncology to environmental studies and oceanography [2, 3].

FC plays a key role in diagnosis of immunological disorders, including HIV, as well as in cancer research [4]. When used in a diagnostic role, cytometry analysis is typically performed on patient blood or bone-marrow samples. The subsequent data-processing analysis is usually done manually, by delineating various cellular populations using 2-D scatter plots and reporting the presence or absence of cellular populations and the proportional composition of the sample [5].

Recently there have been a number of attempts to automate the tasks of interpreting FC measurements [6–14]. Although the methods published vary in their underlining philosophy, the prevailing strategy offered by the researchers cited is strikingly consistent. The algorithms propose various custom modifications of state-of-art clustering techniques ranging in complexity from k-means to mixture modeling.

However, with the exception of the recent report by Cron et al. [15], the published methodology attempts to perform clustering one sample at a time, ignoring the fact that multiple samples can be considered as different realizations of a single underlying model reflecting the biological reality. For samples containing abundant and well-separated biological populations this limitation is of no practical consequence. The individual samples may be clustered, and the biological populations present in multiple samples can be aligned and matched post-clustering in order to perform a secondary analysis (such as longitudinal studies, or comparison of multiple experiments). Multiple efficient methods have been proposed to accomplish this task [16–18].

This conventional approach will fail if some of the cellular classes are represented by a low number of cells, if the population locations significantly vary from sample to sample, or if populations disappear or appear between samples. Indeed, researchers have offered ingenious methods to alleviate some of these problems. For instance, Azad et al. developed a procedure for matching corresponding clusters across samples in order to produce meta-clusters and to construct a high-dimensional template as a collection of meta-clusters for each class of samples [17].

Thus, one of the major problems in the characterization of FC samples is the identification of global, biologically relevant clusters (meta-clusters) corresponding to distinct cell types. Existing methods can be adapted to this problem in two different ways. First, all sample data can be pooled before an algorithm is used to cluster these data. Subsequently, the cell proportions in the recovered clusters may be used to characterize individual samples. flowPeaks [14], FLOCK [19], flowMeans [20], SWIFT [21], and DPGMM [22] are among many clustering techniques that belong in this category. Such an approach will have limited success with many real-world FC data because in the presence of random effects, local clusters belonging to a given global cluster may significantly overlap local clusters of another meta-cluster. As a result, the meta-clusters recovered this way are unlikely to possess a well-defined biological meaning.

Alternatively, a technique such as FLAME that performs joint cell clustering and cluster matching can be used for sample characterization [9]. FLAME first identifies local clusters in each individual sample and then matches them across samples to recover meta-clusters. Although this approach may indeed perform better compared to the first set of techniques that operate on pooled data, cluster matching in the presence of random effects will remain a big challenge. As a result, extraneous clusters may be generated and global clusters corresponding to distinct cell types may be split into multiple sub-clusters. These extraneous clusters appear as feature noise during sample characterization, affecting the robustness of the system.

### Hierarchical clustering models

An alternative model can be envisioned for processing large collections of FC samples. Instead of considering every sample as a separate entity, we explicitly model samples as being specific manifestations of a more general underlying model. In this hierarchical setting, the individual sample is just a noisy realization of a latent, more general biological population mixture. This reformulation has more than just a semantic consequence. It allows us to build a statistical model that takes under consideration all the available information simultaneously, rather than building a single independent model for every sample.

Herein, we present a non-parametric Bayesian algorithm called ASPIRE (anomalous sample phenotype identification with random effects) that identifies biologically significant phenotypes across a batch of samples in the presence of random effects. We do not assume *a priori* the number of cell types (global clusters or meta-clusters) present in the biological samples analyzed, whether they are normal or anomalous. We assume, however, that samples share common characteristics, as they represent snapshots of the same underlying biological phenomenon (e.g., response of the immune system to an external stimulant). Therefore, we expect that certain cell types would occur in multiple samples, forming noisy realizations of global clusters. Our goals are (1) to infer the most likely organization of cell clusters defining normal samples and (2) to detect the presence of anomalous samples.

A related, although simpler, approach has been presented recently by Cron et al. [15]. The authors utilized a hierarchical version of a Dirichlet-process Gaussian-mixture model (DPGMM), extending their previous work [23]. Our proposed approach also belongs to the category of non-parametric Bayesian models using Dirichlet processes. However, in contrast to the method offered by Cron et al. we explicitly model random effects to allow for sample-to-sample variability and subject-specific effects. We provide a complete mathematical framework allowing other researchers to use our methodology, as well as Matlab and C code demonstrating in practice the implementation of the technique.

### Anomaly detection

The presented results demonstrate that the hierarchical model with random effects is superior to traditional per-sample clustering techniques such as FLAME, flowPeaks, and DPGMM as well as to the hierarchical model proposed by Cron et al. In our report we specifically focus on the area of anomaly detection, which is rarely addressed in a systematic manner in the field of cytometry.

An anomaly detection process is extremely difficult to automate using traditional sample-clustering methods. However, an automated anomaly-detection system would provide practical value for computer-aided diagnostics. The majority of results observed in clinical FC are considered "normal," and detecting relatively rare "anomalous" samples requires the immense experience and practice of a well-trained FC practitioner (typically an immunologist or a pathologist).

By dictionary definition an "anomaly" is an oddity or abnormality, hence a case difficult or impossible to classify into any predefined category. In the context of clinical FC data analysis a sample is considered to be anomalous if the phenotypes that it represents do not conform with those expected in the case of a healthy patient. Thus, a sample obtained from a sick patient would be labeled as anomalous. Obviously there could be many possible abnormalities, resulting in a possibly very large number of phenotypic manifestations. Moreover, if a FC measurement is perturbed by the presence of artifacts due to instrument errors or by biological sample-processing or handling errors, the results would also be recognized as anomalous. Consequently, anomalous samples can be as different from each other as they are from normal cases. Although from the biological perspective anomalous cases are extremely important and carry significant biological information, from the machine-learning perspective these samples typically offer only very limited informational value. Because of their rarity it is difficult, and often completely impossible, to model them.

The challenging setting of the anomaly detection framework limits the applicability of traditional supervised methods. A training set may contain a large number of normal cases and just a few anomalous cases, each of which is different from the others. Additionally, those anomalous samples may not be representative for a large and heterogeneous landscape of possible abnormalities. In the context of FC anomaly detection, our technique can be considered semi-supervised as it uses normal samples containing known (predefined or labeled) cell types in order to recognize anomalous samples that may contain additional unknown, often rare cell types.

## Methods

### Data model

FC measurement allows researchers to characterize individual cells present in a biological sample in terms of the abundance of functional markers, such as surface proteins. A data matrix obtained from a FC system upon sample analysis contains measurements of cells organized in rows. The columns represent so-called cytometric parameters, which are typically fluorescence intensities of labels attached to the markers of interest. The data matrix for a typical FC sample may comprise several thousand to a million cells (rows in a matrix), and several FC parameters (columns).

Each biological sample contains multiple, functionally distinct cell types, or "cell populations" in FC vernacular. These populations form multidimensional clusters in the space defined by measured biological features (FC parameters). Although the characteristics (size and multidimensional arrangement) of cell populations present in normal samples are generally known, the number of populations and the proportions of cells present in them could be substantially different in anomalous samples.

We model the data from each sample by a mixture of a potentially infinite number of Dirichlet-process Gaussian-mixture models (DPGMMs), with each individual DPM modeling the local distribution of a single class. Under fairly weak assumptions and given enough components, finite mixtures of Gaussian distributions can model any given density arbitrarily closely [24]. The DPGMM itself is a mixture of a potentially infinite number of Gaussian distributions, with the actual number of mixture components determined directly from the data during inference. Thus, modeling local class distributions by DPGMMs offers the flexibility needed to accommodate skewed or multi-modal distributions. In this context global clusters or meta-clusters refer to functional cell populations, and local clusters or local distributions refer to local realizations of global clusters (cytometry cell populations found in individual samples). Each local cluster is modeled by a DPGMM, i.e., a mixture of a potentially infinite number of Gaussian distributions.

We introduce dependencies across multiple samples by placing a hierarchical DP prior over the base distributions of individual DPGMM models [25]. This hierarchical prior provides a sharing mechanism across samples and allows for sharing of global mixture components across different samples. In FC data analysis, sharing models across multiple samples is a desirable property: a hierarchical prior captures the underlying biological pattern manifested across multiple samples.

We also recognize that limited precision of FC instruments as well as natural biological variability may affect the reproducibility of FC measurements. Therefore, we expand the DPGMM model by postulating the presence of random effects. To account for various sources of sample-to-sample heterogeneity we presume that local cell clusters (relevant populations in the immunophenotypic sense) are generated from noisy versions of corresponding global clusters. Inspired by the random-effects model introduced by Kim and Smyth, we address the random effects by probabilistically modeling the deviations of local cluster means from the means of corresponding global clusters [26].

We provide the technical details of our data model in four stages, following the increasing complexity. In the first stage, we assume that each sample is modeled by a single DPGMM and that DPGMMs across multiple samples are independent. In the second stage, we introduce dependencies across DPGMMs and impose exact sharing of mixture components corresponding to classes across samples. This is equivalent to the HDPGMM model by Cron et al. [15]. In the third stage, we tackle the random effects problem by relaxing the exact sharing of mixture components, allowing local clusters to inherit noisy realizations of classes in individual samples. This approach is equivalent to the HDPGMM-RE model by Kim and Smyth [26]. Finally, in the fourth stage we describe our proposed ASPIRE framework, which models each sample by a potentially infinite mixture of DPGMMs.

#### Independent modeling of samples

As mentioned above, the ASPIRE algorithm models each sample by a DPGMM, a Gaussian-mixture model (GMM) with a Dirichlet-process (DP) prior defined over mixture components [22, 27]. The traditional approach to fitting a Gaussian mixture model onto the data involves using the well-known expectation-maximization algorithm to estimate component parameters [28]. The major limitation of this technique is the need to define the number of clusters in advance. Although there are several ways to estimate the number of clusters in an off-line manner, these methods are in general suboptimal as they decouple two interdependent tasks: predicting the number of clusters and predicting model parameters.

Unlike traditional mixture modeling, DPGMM predicts the number of clusters across multiple samples while simultaneously performing model inference. A DP prior belongs to a group of non-parametric Bayesian models. It is considered "non-parametric" because the number of clusters can arbitrarily grow as needed to accommodate the data. However, the DP prior contains other parameters, the first of which is the precision parameter controlling the prior probability of producing a new cluster and thus indirectly influencing the total number of clusters. The second parameter – the base distribution – defines the Bayesian aspect of the DPGMM. One can utilize the base distribution to encode the existing knowledge of the domain by defining prior distributions over the mean vectors and covariance matrices of components.

We denote cell *i* in sample *j* by ***x***_*ji*_ ∈ ℜ^*d*^, where *i* = {1,…,*n*_*j*._} and *j* = {1,…,*J*}, *n*_*j*._ is the number of cells in sample *j*, and *J* is the total number of samples. In the DPGMM model ***x***_*ji*_ is associated with a mixture component defined by *θ*_*ji*_ = {*μ*_*ji*_,Σ_*ji*_}, which in turn is generated i.i.d. from a DP as follows:
1

*G*_*j*_ are random probability measures distributed i.i.d. according to a DP with a base distribution *G*_0_ and a precision parameter *α*.
2

Using the stick-breaking construction according to Ishwaran and James [29], we can express *G*_*j*_ as  where , , and *ψ*_*jt*_ ∼ *G*_0_. The points *ψ*_*jt*_ are called the *atoms* of *G*_*j*_. Note that unlike a continuous distribution, the probability of sampling the same *ψ*_*jt*_ twice from *G*_*j*_ is not zero and is proportional to *β*_*jt*_. Thus, *G*_*j*_ is considered a discrete distribution and offers a clustering property, as the same *ψ*_*jt*_ can be sampled for different *θ*_*ji*_. In this model *α* is the parameter that controls the prior’s probability of assigning a cell to a new cluster and thus plays a critical role in determining the number of clusters generated.

For the base distribution *G*_0_, from which *ψ*_*jt*_ are drawn, we define a bivariate prior:
3

where ***μ***_0_ is the prior mean and *κ*_0_ is a scaling constant that controls the deviation of the cluster means from the prior mean. The smaller the *κ*_0_, the larger the separation between the cluster means. The parameter Σ_0_ is a positive definite matrix that encodes our prior belief about the expected Σ, i.e., . The parameter *m* is a scalar that is negatively correlated with the degrees of freedom. In other words, the larger the *m*, the less Σ will deviate from *E*(Σ), and vice versa.

#### Introducing dependencies across samples

In the previous section we introduced a clustering property across cells in an individual sample by placing a DP prior over *G*_*j*_ as in Equation (2). Since *G*_*j*_ is a discrete distribution, this prior enables sharing of the same cluster parameter by different cells. When dealing with multiple samples a higher level of sharing occurs. Each local cluster in an individual sample is associated with a global cluster (meta-cluster) representing a specific cell phenotype. Thus, as we cluster cells in each sample, we also group local clusters into appropriate meta-clusters. This grouping can be achieved by placing a hierarchical DP prior over *G*_0_, which introduces dependencies across individual DPGMMs. The hierarchical DPGMM (HDPGMM) for cell clustering and cluster matching across multiple samples becomes
4

where *γ* is the precision parameter for the higher-level DP prior and *H* has the same form as in (3).

Using the stick-breaking construction we can express *G*_0_ as , where , , and *ϕ*_*k*_ = {***μ***_*k*_,Σ_*k*_} ∼ *H*. With this update, instead of letting *G*_0_ be distributed according to (3) as in the independent modeling of samples, we let *H* be distributed according to (3), and let the atoms of *G*_0_ be distributed according to *H*. The distinct set of parameters *ϕ*_*k*_ corresponding to global clusters is sampled from *H* and local cluster parameters are sampled from *G*_*j*_. Since *G*_*j*_ is a discrete distribution with its atoms sampled from *G*_0_, and *G*_0_ is a discrete distribution with its atoms sampled from *H*, each local cluster in turn inherits one of the *ϕ*_*k*_, i.e.,  and , where *K* is the number of global clusters and *m*_*j*._ is the number of local clusters in sample *j*.

Therefore, this model not only groups data points (representing cells) within each sample into clusters, but also groups the local clusters across samples into global clusters (meta-clusters). In other words, clustering and cluster matching are simultaneously addressed and depend on one another.

#### Modeling random effects

In the standard HDPGMM the same parameters are inherited by all local realizations of a global cluster. However, owing to potential random effects caused by biological variability and limited instrument precision this simple framework may be unrealistic. Therefore, to account for random effects we further presume that sample data are generated by noisy versions of the parameters defining global clusters. This change can be incorporated into the data model by updating the model in (4) as follows:
5

where  is a discrete distribution whose atoms are noisy versions of the corresponding atoms in *G*_0_. With this correction to the model each individual sample now inherits different noisy realizations of global parameters, i.e., .

### Modeling individual sample data with multiple DPGMMs

Both HDPGMM and HDPGMM-RE assume that local distributions of classes can be closely approximated by a single Gaussian distribution. This assumption is quite restrictive for many practical settings, as local class data, which are produced subject to random effects, may emerge in the form of skewed as well as multi-mode distributions. As a result, fitting a single Gaussian distribution for local class distributions creates artificial classes that may not be easily distinguished from other significant classes.

ASPIRE uses a potentially infinite mixture of DPGMMs to model each sample’s data, where individual DPGMMs are linked together through a hierarchical DP prior. This hierarchical prior not only identifies local DPGMMs associated with the same class through sharing of a global parameter, but also models the specific subset of classes present and their proportions in each sample.

We update our indexing notation and introduce an additional subscript *k* to account for multiple DPGMMs in each sample. We denote point *i* of class *k* in sample *j* by ***x***_*jki*_ ∈ ℜ^*d*^, where *i* = {1,…,*n*_*jk*._}, *k* = {1,…,*K*}, and *j* = {1,…,*J*}, *n*_*jk*._ is the number of points from class *k* in sample *j*, *K* is the total number of classes, and *J* is the total number of samples. The proposed ASPIRE data model becomes
6

where *ϕ*_*k*_ are global parameters each of which is associated with a different class. Individual DPGMMs associated with the same class inherit the same *ϕ*_*k*_ across samples. The notation  indicates a distribution *F* centered at *ϕ*_*k*_ and defines class-specific base distributions of individual DPGMMs. Although  is same for all DPGMMs associated with the same class, local clusters between samples are generated i.i.d. given *ϕ*_*k*_ of corresponding DPGMMs. Thus, each local realization of a given class is modeled by a different DPGMM, allowing us to account for sample-to-sample variations in a systematic manner.

For the sake of simplicity and to preserve conjugacy we assume that the covariance matrices of all local clusters associated with the same class are identical and limit the susceptibility of local clusters to noise with their mean vectors. More specifically, *μ*_*jki*_ ∼ *G*_*jk*_, Σ_*jki*_ = Σ_*k*_, and  is defined as
7

Note that the covariance matrix of the base distribution  is a function of Σ_*k*_; hence conjugacy of the model is preserved. Conjugacy of the model is important since it enables us to implement a collapsed version of the Gibbs sampler as discussed in the next section. The scaling constant *κ*_1_ adjusts the degree of deviation of local means from the corresponding global mean. A smaller *κ*_1_ results in a situation where local realizations of global means deviate significantly from one sample to another, suggesting significant random effects. On the other hand, a larger *κ*_1_ value limits these deviations, resulting in few to no random effects.

### Model inference

Posterior inference for the proposed model in (6) can be performed by a Gibbs sampler by iteratively sampling local-cluster indicator variables , class indicator variables , and local-cluster parameters  given the state of all other variables. Including ***ψ*** in the Gibbs sampler significantly increases the size of the state space and severely retards the convergence of the Gibbs sampler to the equilibrium distribution. Fortunately, our model uses a conjugate pair of *H* and *p*(·|*ψ*_*jkt*_), which allows us to integrate out *ψ*_*jkt*_ analytically. Thus, we omit the discussion of sampling of ***ψ*** and describe the sampling process for ***t*** and ***c*** only.

When sampling the local-cluster indicator variable *t*_*jki*_ for ***x***_*jki*_ we first remove ***x***_*jki*_ from its current cluster and update the corresponding predictive distribution . Then, we evaluate the likelihood of ***x***_*jki*_’s belonging to an existing cluster by computing  for all local clusters associated with global cluster *k* in sample *j*, and its likelihood of originating from a new cluster by finding the predictive distribution for an empty cluster, i.e., *p*(***x***_*jki*_). Finally, we sample *t*_*jki*_ based on the normalized values of the product of prior probabilities and the corresponding likelihood values. This can be expressed by the following equation:
8

where ***t***^-*jki*^ is the set of all cluster indicator variables, excluding the one for point *i* of class *k* in sample *j*, *D*_…_ denotes the set of all points across all samples,  denotes the subset of points sharing class *c*_*jkt*_ across all samples,  denotes the subset of points in sample *j* belonging to cluster *t* of class *k*, excluding point *i*, *m*_*jk*_ is the number of clusters associated with class *k* in sample *j*, and  is the number of data points in cluster *t* of class *k* in sample *j*, excluding point *i*.

As we model local clusters by Gaussian distributions with Gaussian and inverted Wishart priors defined over their mean vectors and covariance matrices, respectively, the predictive distribution  turns out to be in the form of a Student-t distribution, the derivation of which is provided in Additional file 1.

When sampling the class indicator variable *c*_*jkt*_ for cluster *t* of class *k* in sample *j* we remove points *D*_*jkt*_ from  and update the parameters of the predictive distribution for class *c*_*jkt*_. Then, we evaluate the joint likelihood of cell data in *D*_*jkt*_ for existing classes as well as for a new class. Finally, we sample *c*_*jkt*_ based on the normalized values of the product of prior probabilities of classes and the corresponding joint likelihood values. This can be expressed by the following formula:
9

where  denotes the subset of points across all samples associated with class *k*, excluding points in cluster *t* in sample *j*. The predictive distribution *p*(*x*|*D*_.*k*._) is also in the form of a Student-t distribution and can be readily obtained from  by setting *D*_*jkt*_ an empty set.

Sampling both *t*_*jki*_ and *c*_*jkt*_ requires evaluating the predictive distribution for a new, i.e., an empty, cluster. The predictive distribution for a new cluster is denoted by *p*(***x***_*jki*_) in (8) and (9). This distribution can be obtained from *p*(*x*|*D*_.*k*._) by setting *D*_.*k*._ an empty set.

During a single run of the ASPIRE algorithm one sweep of the Gibbs sampler involves two main iterative loops. In the first loop, *t*_*jki*_ are sampled for all points across all samples. In the second, *c*_*jkt*_ are sampled for all local clusters across all samples. The Gibbs sampler is run for thousand sweeps. The first 750 sweeps are ignored as burn-in, and five samples drawn one every 50 sweeps are saved for final evaluation. Herein we used an approach similar to the one proposed in Cron et al. to deal with label switching [30]. The mode of cluster labels computed across five samples is assigned as the final cluster label for each data instance.

As the first loop iterates over all cell data across all samples it is far more computationally expensive than the second loop. Fortunately, during the sampling process involving *t*_*jki*_ global cluster parameters are fixed. This allows us to sample *t*_*jki*_ independently for each sample during a single sweep and leads to significant improvement in processing time on multi-processor machines. For FC data containing 359,000 cells across 359 samples, the current version of the ASPIRE algorithm implemented in C++ runs in less than thirty minutes on an eight-core computer.

#### Strategy for tuning model parameters

The ASPIRE model has seven free parameters (*α*,*γ*,Σ_0_,*m*,*κ*_0_,*μ*_0_,*κ*_1_), each reflecting a different aspect of the underlying data-generating process. Although data sets resulting from a wide range of experimental settings can be more flexibly modeled by tuning these parameters, an excessive number of free parameters increases the risk of overfitting in addition to affecting the computational time of model optimization. The following describes our strategy to tune these parameters effectively.

As the sample batch may contain anomalous samples, prior information about the potential number of local and global clusters may not exist for most real-world FC data. Thus, for *α* and *γ* we use vague priors by fixing their value to 1. We set *m* to the minimum feasible value, which is *d* + 2, to achieve the maximum number of degrees of freedom. By doing this we let the actual covariance matrices of local and global clusters deviate significantly from the expected covariance matrix, which is . The prior mean *μ*_0_ is set to the mean of the entire data. The scale matrix Σ_0_ is set to *I*/*s*, where *I* is the identity matrix and *s* is a scaling constant.

This leaves *κ*_0_, and *κ*_1_, and the scaling constant *s* of Σ_0_, as the three free parameters that require tuning. We used the FlowCAP 2010 competition lymphoma dataset [12] to tune *s* and *κ*_0_ values empirically. The remaining parameter *κ*_1_ is selected from the set of {0.05, 0.1, 0.25, 0.5, 1} to optimize Gibbs likelihood, which is measured by the joint sampling likelihood of all data points.

### One-class classification by resampling

Once global clusters are identified we can derive a feature vector of global-cluster proportions characterizing each sample. These feature vectors are used for training and testing a one-class classifier. We used the resampling technique to train the classifier [31]. In this approach, a large number of samples is uniformly drawn from the support of the data distribution and all these samples are considered as "positive". Normal cases are considered as "negative". A binary classifier is trained to separate the positive samples from the negative ones.

In the described setting, each sample is characterized by a feature vector of global-cluster proportions whose elements add up to one. If *K* denotes the number of global clusters and *p*_*jk*_, *k* = {1,…,*K*} is the proportion of component *k* in sample *j*, the support of such a data distribution is confined to a simplex of the form 0 ≤ *p*_*jk*_ ≤ 1, . Uniform sampling from this simplex is equivalent to drawing samples from a *k*-variate Dirichlet distribution with all its parameters set to one.

We draw 50,000 samples this way and use this set as the positive class. The feature vectors of normal cases are set as the negative class. Using these data as a training data set we optimize a binary support vector machine (SVM) with a linear kernel and evaluate this classifier on test data containing both normal and anomalous cases. The cost parameter of SVM is tuned by a hold-out approach using a subset of the training data set as a validation set.

## Results and discussion

### Benchmark techniques

In order to evaluate ASPIRE and compare it to state-of-art approaches, four other techniques were experimentally tested for the purpose of this study: conventional DPGMM [22], flowPeaks [14], FLAME [9], and HDPGMM recently published by Cron et al. [15].

Although both DPGMM and flowPeaks are more suitable for clustering single-sample data, they can be used in a batch setting by clustering data pooled from all samples. Using this approach, global clusters can be readily identified without the need for clustering individual samples, finding local clusters, and matching them with one another. Local proportions of global clusters recovered this way can then be used to characterize biological samples. Among many algorithms that can cluster FC data in a pooled setting, DPGMM was our preferred benchmark choice because it originates from the same family of non-parametric Bayesian models as does ASPIRE. The flowPeaks algorithm is also a highly relevant method, as it has recently shown great promise not only in clustering [14] but also in classification of FC samples in a supervised setting [12].

DPGMM fits a Gaussian mixture model with a potentially infinite number of components onto pooled data, with the number of actual components determined during model inference. As each component in the DPGMM is Gaussian, non-Gaussian clusters in the pooled data are unlikely to be captured as a single cluster by DPGMM. ASPIRE is conceptually different from DPGMM – instead of fitting a single Gaussian mixture on pooled data, ASPIRE fits one Gaussian mixture for each individual sample and treats these individual mixture models as noisy realizations of a latent global mixture model.

The flowPeaks algorithm initially partitions data into a large number of clusters using the k-means algorithm and then merges the clusters by exhaustively searching local peaks. Although this agglomerative process in flowPeaks makes capturing non-Gaussian clusters possible, it has significant limitations in the presence of random effects; when the locations of local clusters in the feature space deviate from one sample to another, it is unrealistic to expect all local clusters within a meta-cluster to have a single peak in the pooled data.

Unlike DPGMM and flowPeaks, which cannot perform cluster matching, joint clustering and cluster matching is possible with the FLAME and HDPGMM models.

FLAME fits onto the data from each sample a mixture model with four possible choices of density functions (Gaussian, skewed-Gaussian, t-distribution, skewed-t-distribution) available for individual mixture components. Local modes are pooled and then clustered to obtain a global template of meta-clusters. Local clusters are then assigned to these meta-clusters using graph-matching techniques. FLAME is similar to ASPIRE in the sense that both techniques model individual sample data by a mixture model. However, there are significant differences in model learning. FLAME divides model learning into three tasks: clustering data in individual samples, finding the optimal number of local clusters in each sample, and matching local clusters across samples to recover global clusters. These three tasks are performed by FLAME independently and in a sequential manner.

Unlike FLAME, model learning by ASPIRE is performed as a single unified process. Thus, ASPIRE can take advantage of recurring patterns of similarities across samples. For example, groups of isolated cells forming rare populations that would be ignored as outliers by clustering followed by cluster matching can be successfully identified as rare populations when these two tasks are performed simultaneously. Model learning aside, the major limitation of FLAME occurs when anomalous samples are present in the data set. The FLAME algorithm clusters local modes to generate a template of meta-clusters. This template is unlikely to capture global clusters unique to cell types in anomalous samples, as many of the local modes will be isolated and will likely be clustered with local modes from one of the more dominant cell types.

The HDPGMM by Cron et al. is similar to ASPIRE in certain ways. Both HDPGMM and ASPIRE model individual sample data by a DPGMM and link different DPGMM models through a hierarchical prior. Thanks to the non-parametric nature of these models, the number of local and global clusters can arbitrarily grow in both to accommodate data as needed. Despite these similarities, however, there are important conceptual and algorithmic differences.

The model by Cron et al. does not recognize the presence of random effects and assumes that local clusters are exact realizations of global clusters. In the presence of random effects this assumption is not realistic and leads to the creation of several extraneous global clusters. Cron et al. tackle this problem by post-processing the results to combine global clusters sharing a common mode [15]. This step is very similar to the mode-clustering technique described above for FLAME and relies on two important assumptions: (1) local clusters of a given global cluster share the same mode, and (2) each global cluster has several local realizations. The first assumption is not realistic when random effects are present. The second is not realistic when the data set contains anomalous samples characterized by isolated, phenotypically abnormal cell types.

Unlike HDPGMM, ASPIRE assumes that local clusters are noisy realizations of global clusters, and probabilistically models the deviations of the local cluster means from the corresponding global cluster means. As random effects are already taken into account during model learning, no post-processing is required with ASPIRE. Apart from these conceptual differences there are also algorithmic differences between ASPIRE and HDPGMM. The state space of HDPGMM contains cluster parameters. This slows down convergence of the sampling process. ASPIRE uses a conjugate data model that makes possible the implementation of a collapsed Gibbs sampler As a result, the state space of the ASPIRE model does not contain cluster parameters. Eliminating cluster parameters from sampling speeds up convergence.

For testing FLAME performance we used the version implemented in GenePattern [32]. When running the FLAME algorithm for the simulated data, we fit each sample by a Gaussian mixture model since the cluster data were generated by Gaussian distributions. For the real FC data, we fit each sample by a skewed-t mixture model as suggested in the original FLAME report [9]. For HDPGMM testing we used the software provided by the authors [15]. The flowPeaks algorithm was tested using the *R* version available through Bioconductor [33]. The DPGMM method was tested utilizing our own implementation of the algorithm by Cron et al. The performance of the models tested is evaluated by the area under the receiver operating characteristics (ROC) curve, in short the AUC value.

### Experiment 1: synthetic data containing three types of anomalies

The purpose of this *in silico* experiment is to illustrate the ability of ASPIRE to recover global clusters and identify anomalous samples. We generated twenty-five samples, each with one thousand data points in a two-dimensional feature space. Ten of the samples were considered normal, and global clusters associated with samples in this group were treated as normal clusters. Fifteen anomalous samples were generated in three groups, each group simulating a different anomalous effect.

The samples in the first group were associated with the same set of global clusters found in the normal samples. An anomaly effect was produced by creating samples with different relative proportions of points belonging to these clusters.

The generative model associated with samples in the second group involved six global clusters, three of which were normal clusters and another three of which contained different rare populations. Each rare population consisted of only five data points. The relative proportions of points in the normal clusters were the same as those in normal samples.

**Figure 1:**
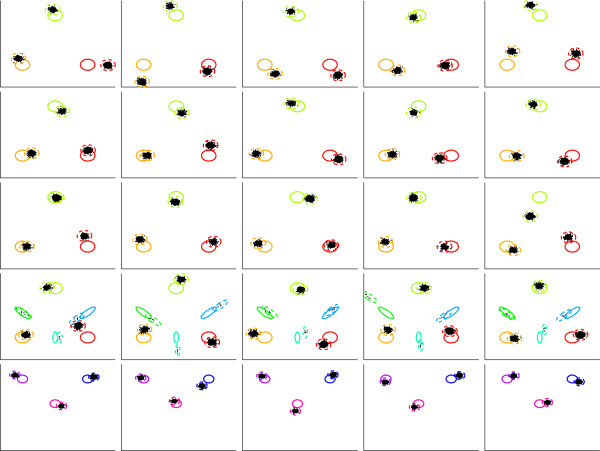
**Global and local clusters for twenty-five simulated samples.** Plots in the top two rows correspond to normal samples. Rows three through five show plots of anomalous samples produced by introducing rare populations or by distorting distributions of normal clusters. Solid and dashed ellipses indicate distribution of global and local clusters, respectively. Individual instances are shown by black points. Distributions sharing the same global cluster (meta-cluster) across different samples are identified by the same color.

The samples in the third group were generated to simulate measurement artifacts. To achieve this effect, three global clusters were derived from normal clusters by shifting their mean vectors and reducing standard deviations in each dimension by half. To introduce random effects into the generative process the points in each sample were generated from the local clusters using *κ*_1_ = 0.05. Distribution of global and local clusters and the data points corresponding to events for all twenty-five samples are shown in Figure 1. Ellipses in the figures correspond to data distributions that are at most four standard deviations from the mean.

**Figure 2:**
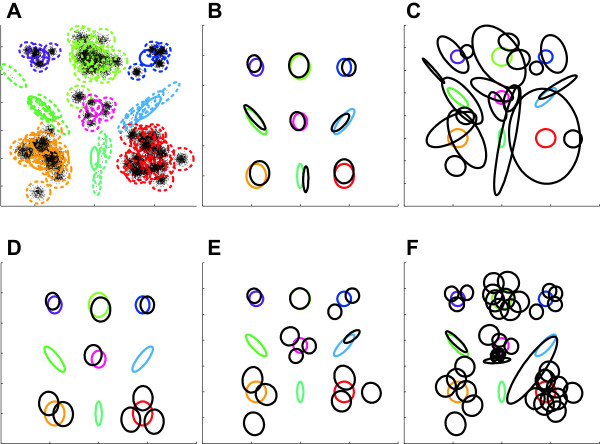
**Distributions recovered by competing techniques.** Solid- and dashed-color ellipses indicate global and local clusters, respectively. Solid-black ellipses show recovered distributions of global clusters. **A**. Pooled data. **B**. ASPIRE. **C**. DPGMM. **D**. FLAME. **E**. FlowPeaks. **F**. HDPGMM.

We applied all five techniques (ASPIRE, DPGMM, HDPGMM, flowPeaks, and FLAME) to this data set and plotted recovered distributions of global clusters for each case in Figure 2 along with the pooled data from all twenty-five samples. The results demonstrate that all techniques but ASPIRE failed to recover accurately the distributions of global clusters owing to large inter-sample variance affecting local clusters.

ASPIRE not only correctly predicted the true number of global clusters but also recovered their corresponding distributions with fairly good precision. Between the two techniques operating with pooled data (DPGMM and flowPeaks) flowPeaks seemed to handle random effects better as it accurately recovered distributions for two of the nine global clusters. Of the two techniques, other than ASPIRE, operating with individual sample data (HDPGMM and FLAME), HDPGMM suffers significantly from random effects. Although it accurately recovered distributions of local clusters, it failed to consistently match local clusters across samples, and as a result substantially overestimated the actual number of global clusters. Compared to HDPGMM, FLAME performed relatively well and accurately recovered distributions of four of the nine global clusters. However, FLAME failed to process all five samples containing rare clusters. The errors generated during the mixture-modeling stage suggest that the FLAME process cannot properly initialize cluster centers when there are isolated clusters with very few data points. Additional experimental results with ASPIRE investigating the effect of isolated rare classes and limited random effects on simulated data are provided in Additional file 2.

### Experiment 2: purdue healthy subjects (PHS) data set

The PHS data set contains FC results of a 5-parameter blood analysis performed using samples collected from five healthy donors. In each sample five fluorescent labels – PC5, FITC, PE, PC7, and ECD – are used to identify cells expressing CD45, CD4, CD8, CD3, and CD19 markers, respectively.

**Figure 3:**
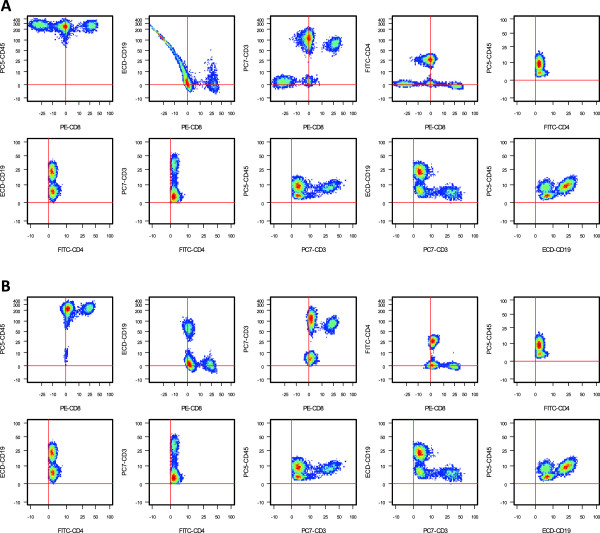
**Examples of a normal and an anomalous sample in the Purdue data set.** 2D scatter plots of cells expressing CD45, CD4, CD8, CD3, and CD19 markers. **A**. Anomalous sample. **B**. Normal sample.

The sample collection and data acquisition were performed over a number of days. In accordance with standard FC data-analysis procedures, samples were pre-processed by performing linear spectral unmixing (compensation) [34, 35]. In order for the compensation to return approximate abundances of the labels used, one must employ the correct spillover matrix obtained from single-stained controls run under identical experimental conditions. However, in post-processing, it was discovered that a small subset of samples had been compensated using the wrong controls. These samples are readily identifiable by trained cytometrists (Figure 3). We consider the improperly unmixed samples to be anomalous. The task for the algorithm was to find the anomalous samples automatically. This task mimics a typical data-quality check step performed on a large collection of flow cytometry data.

We used a total of 81 samples, five of which were anomalous. The data set obtained by subsampling ten percent of the cell data from each sample contained 144,000 data points. Data corresponding to each marker were transformed logarithmically and standardized to have unit variance. With this data set FLAME failed to properly process nine of the normal cases, which were excluded from subsequent analysis of FLAME performance. The other four techniques were evaluated on the entire data set. An anomalous sample along with a typical normal sample is shown in Figure 3. The experimental settings used in experiments for all five techniques are summarized in Additional file 3. The trace plot obtained by ASPIRE is shown in Additional file 4.

**Figure 4:**
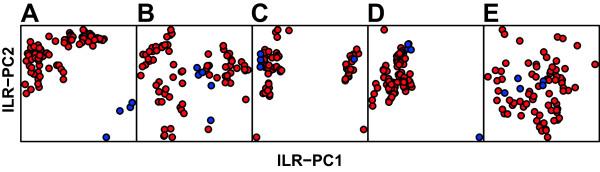
**2D scatter plots obtained by the robust compositional PCA algorithm using cell-type proportions predicted by each of the five algorithms on the Purdue healthy subjects data set with anomalies.** Properly compensated samples are shown by blue circles and those with compensation artifacts by red circles. **A**. ASPIRE. **B**. DPGMM. **C**. FLAME. **D**. FlowPeaks. **E**. HDPGMM.

The numbers of global clusters identified for the PHS set by each of the five algorithms are shown in Table 1. We characterize the samples with feature vectors of global-cluster proportions produced by the five algorithms. Since the feature vectors describe composition of the samples (they sum to one), we visualize the results with 2-D scatter plots representing compositional principal component analysis rather than standard PCA (See Figure 4) [36]. After processing with the ASPIRE algorithm, the anomalous samples are clearly isolated and can be easily identified in Figure 4. For the other four algorithms the distinction between normal and anomalous cases is not obvious.

**Table 1:** **Number of global clusters identified by each algorithm after excluding small clusters containing less than 0.5% of the total number of cells across all samples**

	Purdue	AML tubes						
		2	3	4	5	6	7	All
ASPIRE	12	9	18	21	10	8	17	83
DPGMM	50	49	47	42	56	47	52	283
FLAME	4	-	-	-	-	-	-	-
flowPeaks	4	6	6	10	5	4	7	38
HDPGMM	16	58	68	68	60	80	54	388

Reported values are modes of ten repetitions. FLAME results for the AML data set are not included as FLAME produced errors on many of the samples in this data set.

Next, we used samples from three subjects for training (51 samples) and samples from the remaining two subjects along with five anomalous samples (30 samples) for testing. We trained a one-class Dirichlet-SVM classifier described previously under the Methods section and evaluated it on the test set. We repeated this process ten times, each time with a different set of positive samples drawn from the Dirichlet distribution. AUC values obtained by all five techniques are included in the first column of Table 2. ASPIRE achieved the perfect AUC value. The flowPeaks algorithm and FLAME produced comparable AUC values of 0.94 and 0.93, respectively. DPGMM achieved an AUC value of 0.80. With an AUC of 0.51 HDPGMM cannot compete with the other techniques.

**Table 2:** **AUC values achieved by each algorithm on the Purdue and AML data sets**

	Purdue	AML tubes						
		2	3	4	5	6	7	All
ASPIRE	1.000	0.940	0.974	0.991	0.999	0.992	0.971	0.997
	(0.000)	(0.025)	(0.003)	(0.003)	(0.001)	(0.010)	(0.005)	(0.002)
DPGMM	0.995	0.782	0.612	0.933	0.935	0.954	0.514	0.773
	(0.010)	(0.068)	(0.070)	(0.027)	(0.019)	(0.015)	(0.120)	(0.088)
FLAME	0.930	-	-	-	-	-	-	-
	(0.000)							
flowPeaks	0.944	0.369	0.430	0.982	0.806	0.906	0.670	0.857
	(0.000)	(0.003)	(0.001)	(0.001)	(0.004)	(0.002)	(0.015)	(0.038)
HDPGMM	0.576	0.452	0.493	0.530	0.600	0.571	0.509	0.532
	(0.005)	(0.011)	(0.015)	(0.011)	(0.015)	(0.027)	(0.011)	(0.009)

Numbers in parentheses are standard deviations across ten repetitions of the corresponding one-class classifiers. FLAME results for the AML data set are not included as FLAME produced errors on many of the samples in this data set.

### Experiment 3: DREAM6/FlowCap-II acute myeloid leukemia (AML) data set

This data set, which was originally made available for the DREAM6/FlowCAP-II Molecular Classification of AML Challenge, contains samples from 43 AML-diagnosed patients and 316 healthy patients [12]. Each subject sample was subdivided into 8 tubes and analyzed for the presence of different marker combinations (5 markers per tube). In addition to the five markers, the forward scatter (FS) and side scatter (SS) of each sample were also measured for each tube. We exclude the two control tubes (tubes 1 and 8) and report results on the remaining six. The data for side scatter (SS) and for all the fluiorescent markers were transformed logarithmically, whereas the data for forward scatter (FS) remained linear. Data for each channel are also standardized to have unit variance.

Although the DREAM6/FlowCAP-II contest was designed for traditional supervised classification of AML and healthy cases, we are using this data set in a considerably more challenging setting. Unlike the contest, where participants had access to AML cases during both training and testing, we did not include any AML cases in the training. Instead, we tried to find whether the proposed ASPIRE model can identify anomalies even when they are not defined or demonstrated *a priori*. Our training data set contained samples from 150 normal subjects, whereas the testing data set contained samples from 166 normal subjects and 43 AML-positive patients. The data set for each tube was subsampled to contain 1,000 cells from each sample for a total 359,000 × 6 cell data points across 359 samples.

We first report our results separately for each tube and then report results for the combined data by concatenating feature vectors of global-cluster proportions for the six tubes. The number of global clusters and the AUC values achieved by each technique for individual tubes and for their combinations are included in Tables 1 and 2, respectively. Since the FLAME algorithm produced errors during processing of many samples in the AML data set, no results are reported for FLAME performance on this data set. The experimental settings used in experiments for all five techniques are summarized in Additional file 3. Trace plots obtained by ASPIRE are shown in Additional file 4.

Among the four remaining algorithms tested, ASPIRE achieved the highest AUC values for all individual tubes as well as for the combined data. The AUC values given by ASPIRE exceeded 0.90 for all tubes, with an AUC of 0.99 achieved for three of the six tubes. The average number of global clusters recovered per tube by ASPIRE was 13.8.

DPGMM and flowPeaks produced AUCs above 0.9 for two of the six tubes. The average number of global clusters recovered per tube for these two algorithms was 47.1 and 6.3, respectively. Results obtained by HDPGMM were not promising. The AUC values achieved by HDPGMM suggest that the model did not perform better than random chance for most of the tubes. HDPGMM also produced a large number of extraneous global clusters.

The AUC values obtained by combining data from all the tubes were lower than the maximal AUC values achieved for the individual tubes for all four techniques. This was expected, as feature noise present in the feature vectors describing individual tubes accumulates with concatenation [37]. Among the four techniques ASPIRE suffered the least from this noise effect and showed the least degradation in the maximum AUC value after all the tubes were combined.

Although the main objective of this experiment was to demonstrate that global clusters discovered by ASPIRE are useful for identifying anomalous samples in a one-class classification setting, ASPIRE can also be used in a fully supervised classification setting with both normal and anomalous classes represented in the training data set. To show that ASPIRE is also competitive in a traditional supervised classification setting we followed the procedure adopted in the DREAM6/FlowCAP-II challenge to train and test a supervised classifier. The results in Table 3 suggest that in a supervised mode ASPIRE can match the best-performing techniques listed in the FlowCAP-II report [12].

**Table 3:** **Supervised-classification accuracies for ASPIRE on the AML data set**

	AML tubes						
	2	3	4	5	6	7	All
ASPIRE	96.9	97.7	98.5	99.2	100.0	98.2	98.9
	(0.8)	(1.2)	(0.6)	(0.9)	(0.0)	(1.1)	(0.3)

Numbers in parentheses are standard deviations across ten repetitions.

### Discussion

The unrealistically large number of meta-clusters and poor AUC values generated by HDPGMM suggest that the cluster-matching aspect of this algorithm suffers significant problems with sample heterogeneity. Techniques that operate with pooled data (DPGMM and flowPeaks) performed better compared to HDPGMM in terms of meta-cluster numbers and classification results. Results from all three experiments suggest that flowPeaks tends to underestimate whereas DPGMM typically overestimates the number of global clusters. FLAME positions itself in the middle, seemingly handling the more abundant cell populations well but failing to identify rare cell types. The presence of multiple spurious meta-clusters generated by FLAME in Experiments 1 and 2 indicate that the mode-clustering algorithm employed by FLAME is not very effective in the presence of random effects.

Compared with the benchmark techniques, ASPIRE is more effective in capturing the phenotypic patterns linked with anomalies in biological characteristics. In fact, in experiments with synthetic data (set 1), ASPIRE not only correctly inferred the number of meta-clusters but also identified all the anomalous samples with perfect accuracy. In experiments with real-world FC data ASPIRE produced reasonable numbers of global clusters and achieved almost perfect AUC values (See Table 2).

In terms of computation time (assesed using a single-core computer), flowPeaks – a k-means–based technique – was by far the fastest algorithm. It takes less than a minute to run flowPeaks on the subset of the AML data set containing 359,000 data points across 359 samples (one tube per sample). In contrast, the processing time for a single tube from the AML data set takes about 12 hours for HDPGMM, 3-4 hours for DPGMM, and less than 2 hours for ASPIRE. The processing time for FLAME (including the failed cases) was also close to 12 hours.

The processing time required by HDPGMM and ASPIRE can be significantly reduced by running the algorithms in a multi-core mode. Although we were not able to test the multi-core version of HDPGMM by Cron et al. owing to operating system restrictions, about four-fold improvement in run time was observed for ASPIRE executed on an eight-core machine.

## Conclusions

We introduced ASPIRE as a new method for sample characterization in FC that performs joint cell clustering and cluster matching in the presence of random effects. The algorithm operates in a batch setting, discovering global clusters in collections of FC data. By utilizing a non-parametric clustering approach paired with a hierarchical model, ASPIRE addresses the issue of anomaly detection in a way both unique and original. In contrast to established FC processing techniques, ASPIRE provides higher robustness and the ability to incorporate experimentally acquired notions of biological and technical (instrumental) variability.

The reported experimental results obtained from analyzing synthetic and real data favor ASPIRE over other benchmark techniques considered for anomaly detection. Results also indicate that by modeling potential random effects ASPIRE is able to produce a realistic number of meta-clusters that are interpretable in the biological context. This contrasts with the unexpectedly large number of meta-clusters generated by DPGMM and HDPGMM, the other Dirichlet process–based methods. The impressive AUC values demonstrate the unique capability of ASPIRE to detect and identify anomalous samples in the complete absence of information regarding the characteristics of anomalies. In other words, ASPIRE is able to form a reasonable generalization on the basis of normal cases, and – like experienced cytometrists – use this generalization to locate suspicious and abnormal cases.

In the proposed approach anomalies are detected by a two-stage process involving the discovery of meta-clusters followed by one-class classification of feature vectors of cluster proportions characterizing samples. These two stages can be combined into one by a nested Dirichlet-process model [38] that can cluster not only events and populations but samples as well. Another avenue for future research involves incorporation of partial knowledge about anomaly characteristics. The reported model assumes that anomalies are completely unknown; however, one can envision a setting in which a reasonable approximation of anomaly characteristics can be hypothesized. Our model can account for such a framework by employing a restricted version of the Gibbs sampler.

ASPIRE is implemented in C++ and is available as stand-alone executable software. Matlab^®;^ (Natick, MA) scripts are also provided for using ASPIRE within the Matlab platform. The software is freely available from http://cs.iupui.edu/~dundar/aspire.htm.

## Electronic supplementary material

Additional file 1: **Evaluation of the Predictive Distributions for Local and Global Clusters.** (PDF 143 KB)

Additional file 2: **Additional Experimental Results with Simulated Data.** (PDF 1023 KB)

Additional file 3: **Model Settings used in Experiments with PHS and AML Data Sets.** (PDF 105 KB)

Additional file 4: **Trace Plots of Cluster Proportions in Experiments with PHS and AML Data Sets.** (PDF 467 KB)
