# Supplementary material for: A non-parametric Bayesian model for joint cell clustering and cluster matching: identification of anomalous sample phenotypes with random effects
Source: BMC Bioinformatics. 2014 Sep 24;15(1):314. doi: 10.1186/1471-2105-15-314 (PMC4262223; doi:10.1186/1471-2105-15-314)
Supplement: Supplementary file 1 — Additional file 1: Evaluation of the Predictive Distributions for Local and Global Clusters. (PDF 143 KB) [file 12859_2014_6631_MOESM1_ESM.pdf]

Additional File 1

Evaluation of the Predictive Distributions for Local and Global Clusters

June 23, 2014

1 Evaluation of the Predictive Distributions for Local and Global Clusters

Data Model:

$$\begin{array}{lcl} p(\boldsymbol{x}_{jki}|\boldsymbol{\mu}_{jkt},\Sigma_k) & \sim & N\left(\boldsymbol{\mu}_{jkt},\Sigma_k\right) \\ p(\boldsymbol{\mu}_k|\Sigma_k) & \sim & N\left(\boldsymbol{\mu}_0,{\kappa_0}^{-1}\Sigma_k\right) \\ p(\Sigma_k) & \sim & W^{-1}\left(\Sigma_0,m\right) \end{array} \tag{1}$$

Noise Model:

$$p(\boldsymbol{\mu}_{jkt}|\boldsymbol{\mu}_k,\Sigma_k) \sim N\left(\boldsymbol{\mu}_k,(\kappa_1)^{-1}\Sigma_k\right) \tag{2}$$

In the above equations $\boldsymbol{\mu}_{jkt}$ is the mean of the local cluster t of the global cluster k in sample j, $\boldsymbol{\mu}_k$ is the mean of the global cluster k, Σ_k is the covariance matrix of the global cluster k as well as the covariance matrix of all local clusters sharing global cluster k (no random effects assumed over the covariance matrices), $\boldsymbol{\mu}_0$ is the prior mean and κ_0 is a scaling constant that controls the deviation of the global cluster means from the prior mean, κ_1 is a scaling constant that controls the deviation of the local cluster means from the corresponding global cluster mean, Σ_0 is a positive definite matrix that encodes our prior belief about the expected Σ_k, and m is a scalar that is negatively correlated with the degrees of freedom.

For the data and noise models above the predictive distribution of a local cluster can be derived by evaluating the following integral:

$$p(\boldsymbol{x}_{jki}|D_{.c_{jkt}.},D_{jkt}) = \int\int p(\boldsymbol{x}_{jki}|\boldsymbol{\mu}_{jkt},\Sigma_k)p(\boldsymbol{\mu}_{jkt},\Sigma_k|D_{.c_{jkt}.},D_{jkt})\partial\boldsymbol{\mu}_{jkt}\partial\Sigma_k \tag{3}$$

where $D_{.c_{jkt}.}$ denotes the subset of cells sharing global cluster c_{jkt} across all clusters and D_{jkt} denotes the subset of cells in sample j belonging to local cluster t of global cluster k.

To evaluate the integral in (3) we need the posterior distribution of the parameters $p(\boldsymbol{\mu}_{jkt},\Sigma_k|D_{.c_{jkt}.},D_{jkt})$, which can be expressed as follows using the Bayes theorem:

$$p(\boldsymbol{\mu}_{jkt},\Sigma_k|D_{.c_{jkt}},D_{jkt}) = \frac{p(\boldsymbol{\mu}_{jkt},\Sigma_k,\bar{\boldsymbol{x}}_{jkt},A_{jkt}|\bar{\boldsymbol{x}}_{jkt:c_{jkt}=k},A_k)}{\int\int p(\boldsymbol{\mu}_{jkt},\Sigma_k,\bar{\boldsymbol{x}}_{jkt},A_{jkt}|\bar{\boldsymbol{x}}_{jkt:c_{jkt}=k},A_k)\partial\boldsymbol{\mu}_{jkt}\partial\Sigma_k} \tag{4}$$

where $\bar{\boldsymbol{x}}_{jkt}$ and A_{jkt} are the sample mean and the scatter matrix for the local cluster t of global cluster k in sample j, respectively, and A_k is the scatter matrix for the global cluster k. These statistics are defined as in (5).

$$\begin{array}{lcl} \bar{\boldsymbol{x}}_{jkt} & = & n_{jkt}^{-1}\sum_{jki:t_{jki}=t}\boldsymbol{x}_{jki} \\ A_{jkt} & = & \sum_{jki:t_{jki}=t}\left(\boldsymbol{x}_{jki}-\bar{\boldsymbol{x}}_{jkt}\right)\left(\boldsymbol{x}_{jki}-\bar{\boldsymbol{x}}_{jkt}\right)^T \\ A_k & = & \sum_{jkt:c_{jkt}=k}A_{jkt} \end{array} \tag{5}$$

In order to evaluate (4) we need to obtain $p(\boldsymbol{\mu}_{jkt},\Sigma_k,\bar{\boldsymbol{x}}_{jkt},A_{jkt}|\bar{\boldsymbol{x}}_{jkt:c_{jkt}=k},A_k)$, which can be expanded as follows:

$$\begin{aligned} p(\boldsymbol{\mu}_{jkt},\Sigma_k,\bar{\boldsymbol{x}}_{jkt},A_{jkt}|\bar{\boldsymbol{x}}_{jkt:c_{jkt}=k},A_k) = {} & p(\bar{\boldsymbol{x}}_{jkt}|\mu_{jkt},\Sigma_k)p(A_{jkt}|\Sigma_k) \\ & p(\mu_{jkt}|\Sigma_k,\bar{\boldsymbol{x}}_{jkt:c_{jkt}=k})p(\Sigma_k|A_k) \end{aligned} \tag{6}$$

where

$$\begin{array}{lcl} p(\bar{\boldsymbol{x}}_{jkt}|\Sigma_k) & = & N\left(\mu_{jkt},{n_{jkt}}^{-1}\Sigma_k\right) \\ p(A_{jkt}|\Sigma_k) & = & W\left(\Sigma_k,n_{jkt}-1\right) \\ p(\boldsymbol{\mu}_{jkt}|\Sigma_k,\bar{x}_{jkt:c_{jkt}=k}) & = & N(\bar{\boldsymbol{\mu}},\bar{\kappa}^{-1}\Sigma_k) \\ p(\Sigma_k|A_k) & = & W^{-1}\left(\Sigma_0+A_k,m+\sum_{jkt:c_{jkt}=k}(n_{jkt}-1)\right) \end{array} \tag{7}$$

and $\bar{\boldsymbol{\mu}}$ and $\bar{\kappa}$ are defined as follows:

$$\bar{\boldsymbol{\mu}} = \frac{\sum_{jkt:c_{jkt}=k} \frac{n_{jkt}\kappa_1}{(n_{jkt}+\kappa_1)} \bar{\boldsymbol{x}}_{jkt} + \kappa_0 \mu_0}{\sum_{jkt:c_{jkt}=k} \frac{n_{jkt}\kappa_1}{(n_{jkt}+\kappa_1)} + \kappa_0}$$

$$\bar{\kappa} = \frac{(\sum_{jkt:c_{jkt}=k} \frac{n_{jkt}\kappa_1}{(n_{jkt}+\kappa_1)} + \kappa_0)\kappa_1}{\sum_{jkt:c_{jkt}=k} \frac{n_{jt}\kappa_1}{(n_{jkt}+\kappa_1)} + \kappa_0 + \kappa_1}$$

Once the distributions in (7) are substituted into (4) a closed-form expression for $p(\boldsymbol{\mu}_{jkt}, \Sigma_k | D_{.c_{jkt}.}, D_{jkt})$ can be obtained. When we substitute this solution into (3) we obtain $p(\boldsymbol{x}_{jki} | D_{.c_{jkt}.}, D_{jkt})$ in the form a multivariate student-t distribution with three parameters.

$$p(\boldsymbol{x}_{jki} | D_{.c_{jkt}.}, D_{jkt}) = stu - t(\hat{\boldsymbol{\mu}}, \hat{\Sigma}, v) \tag{8}$$

The location vector $(\hat{\mu})$, the scale matrix $(\hat{\Sigma})$, and the degrees of freedom (v) are given below.

Location vector:

$$\hat{\boldsymbol{\mu}} = \frac{n_{jkt}\bar{\boldsymbol{x}}_{jkt} + \bar{\kappa}\bar{\boldsymbol{\mu}}}{n_{jkt} + \bar{\kappa}} \tag{9}$$

Scale matrix:

$$\hat{\Sigma} = \frac{\Sigma_0 + A_k + A_{jkt} + \frac{n_{jkt}\bar{\kappa}}{n_{jkt}+\bar{\kappa}} (\bar{\boldsymbol{x}}_{jkt} - \bar{\boldsymbol{\mu}})(\bar{\boldsymbol{x}}_{jkt} - \bar{\boldsymbol{\mu}})^T}{\frac{(\bar{\kappa}+n_{jkt})\, v}{(\bar{\kappa}+n_{jkt}+1)}} \tag{10}$$

Degrees of freedom:

$$v = m + \sum_{jkt:c_{jkt}=k} (n_{jkt} - 1) + n_{jkt} - d + 1 \tag{11}$$

The predictive distribution of a global cluster can be readily obtained from $p(\boldsymbol{x}_{jki} | D_{.c_{jkt}.}, D_{jkt})$ by setting D_{jkt} an empty set. This is equivalent to dropping terms related to local clusters in equations (9), (10), and (11).

$$p(\boldsymbol{x}_{jki} | D_{.k.}) = stu - t(\hat{\boldsymbol{\mu}}, \hat{\Sigma}, v) \tag{12}$$

The location vector $(\hat{\mu})$, the scale matrix $(\hat{\Sigma})$, and the degrees of freedom (v) are redefined for a global cluster as follows.

Location vector:

$$\hat{\boldsymbol{\mu}} = \bar{\boldsymbol{\mu}} \tag{13}$$

Scale matrix:

$$\hat{\Sigma} = \frac{\Sigma_0 + A_k}{\frac{\bar{\kappa}\, v}{\bar{\kappa}+1}} \tag{14}$$

Degrees of freedom:

$$v = m + \sum_{jkt:c_{jkt}=k} (n_{jkt} - 1) - d + 2 \tag{15}$$

where $\bar{\boldsymbol{\mu}}$ and $\bar{\kappa}$ are defined as in (8) and (8), respectively.

Finally, the predictive distribution of an empty cluster can be obtained from $p(\boldsymbol{x}_{jki} | D_{.k.})$ by setting $D_{.k.}$ an empty set. This is equivalent to dropping terms related to global clusters in equations (13), (14), and (15).

$$p(\boldsymbol{x}_{jki}|D_{.k.}) = stu - t(\hat{\boldsymbol{\mu}}, \hat{\Sigma}, v) \tag{16}$$

The location vector ($\hat{\mu}$), the scale matrix ($\hat{\Sigma}$), and the degrees of freedom (v) are redefined for an empty cluster as follows:

Location vector:

$$\hat{\boldsymbol{\mu}} = \boldsymbol{\mu}_0 \tag{17}$$

Scale matrix:

$$\hat{\Sigma} = \frac{\Sigma_0}{\frac{\kappa_1 \kappa_0 \, v}{\kappa_1 \kappa_0 + \kappa_0 + \kappa_1}} \tag{18}$$

Degrees of freedom:

$$v = m - d + 2 \tag{19}$$
