# Supplementary material for: A non-parametric Bayesian model for joint cell clustering and cluster matching: identification of anomalous sample phenotypes with random effects
Source: BMC Bioinformatics. 2014 Sep 24;15(1):314. doi: 10.1186/1471-2105-15-314 (PMC4262223; doi:10.1186/1471-2105-15-314)
Supplement: Supplementary file 2 — Additional file 2: Additional Experimental Results with Simulated Data. (PDF 1023 KB) [file 12859_2014_6631_MOESM2_ESM.pdf]

Additional File 2

Additional Experimental Results with Simulated Data

June 23, 2014

1 Additional Experimental Results with Simulated Data

We present additional experimental results using the simulated data from Experiment 1 in the main text. First, we investigated the effect of the number of local realizations of rare classes on ASPIRE performance. We started with a data set in which rare classes appeared only once, i.e., were realized in a single sample, and ran ASPIRE to see if rare classes can be accurately identified in such a setting. We then repeated this experiment, each time increasing the number of realizations of rare classes by one.

Only one of the three rare classes was recovered with a single realization (see Fig. 1). All three rare classes were recovered with two or more realizations (see Figs 2-5). In these figures solid- and dashed-color ellipses indicate global and local clusters, respectively. Solid-black ellipses show recovered distributions of global clusters. These results suggest that when the local distribution of a rare class is well-separated from the rest of the classes ASPIRE can recover that distribution even when the rare class appears only once. When the only realization of a rare class is not well-separated, additional instances might be required.

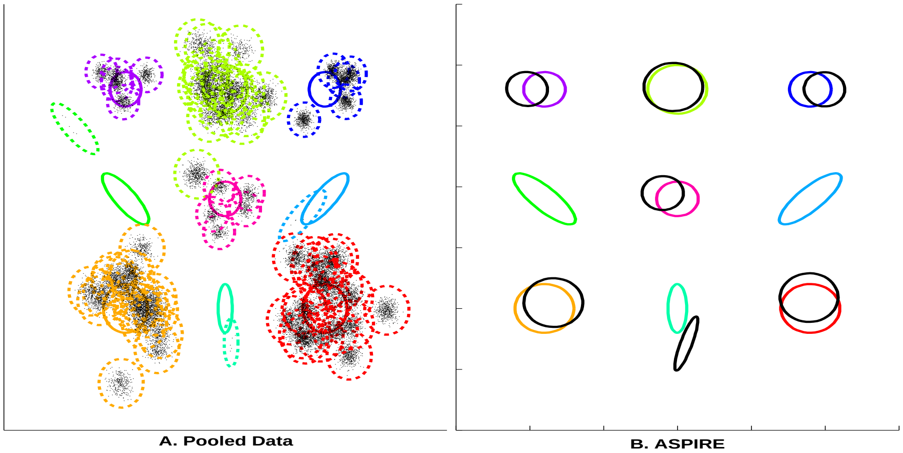

Figure 1: Rare classes with a single local realization.

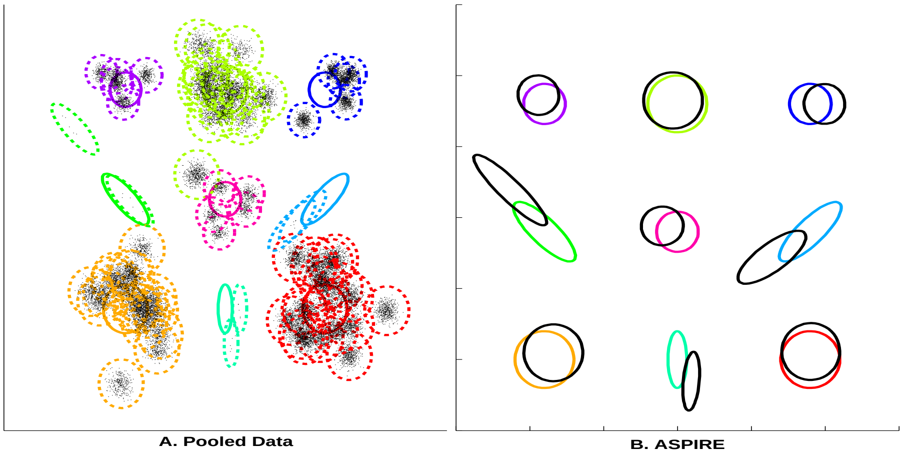

Figure 2: Rare classes with two local realizations.

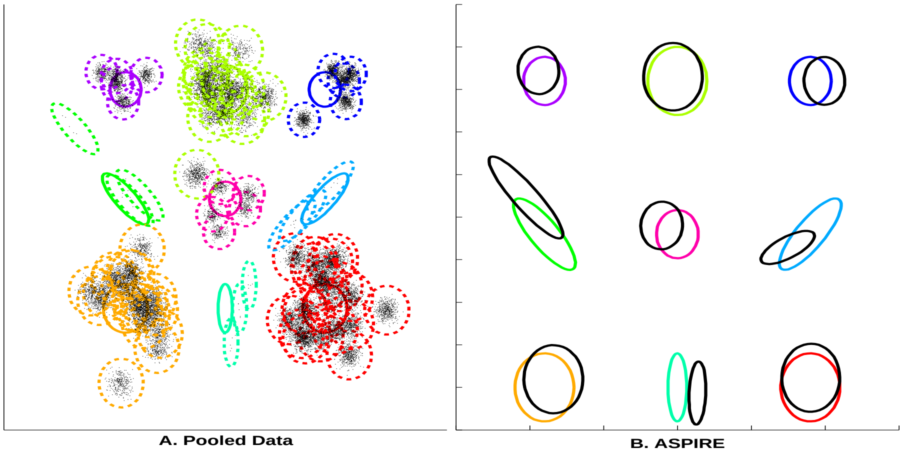

Figure 3: Rare classes with three local realizations.

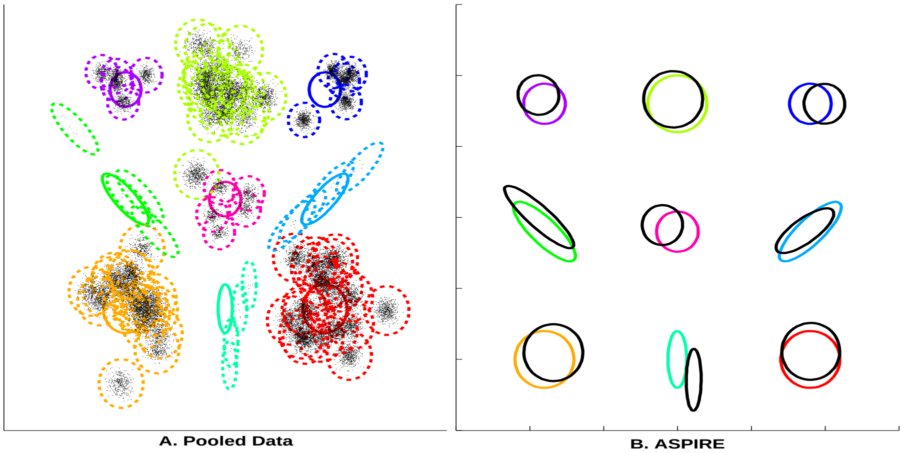

Figure 4: Rare classes with four local realizations.

We also investigated the performance of ASPIRE with a larger κ_1 (0.2) than used in Experiment 1 of the main text (0.05). A larger κ_1 implies smaller deviation of local cluster centers from the global cluster center and produces a relatively easier problem setting for ASPIRE, since fewer random effects would be present. As the plot in Fig. 6 shows, ASPIRE successfully recovered all class distributions under this setting.

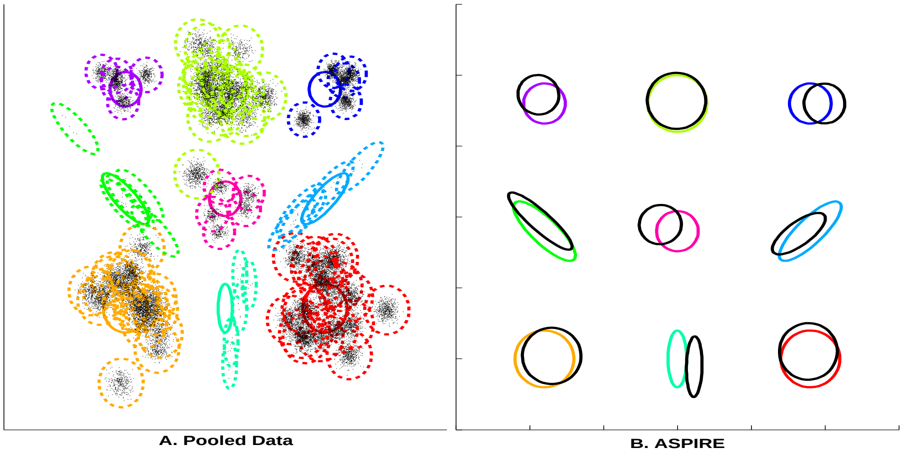

Figure 5: Rare classes with five local realizations.

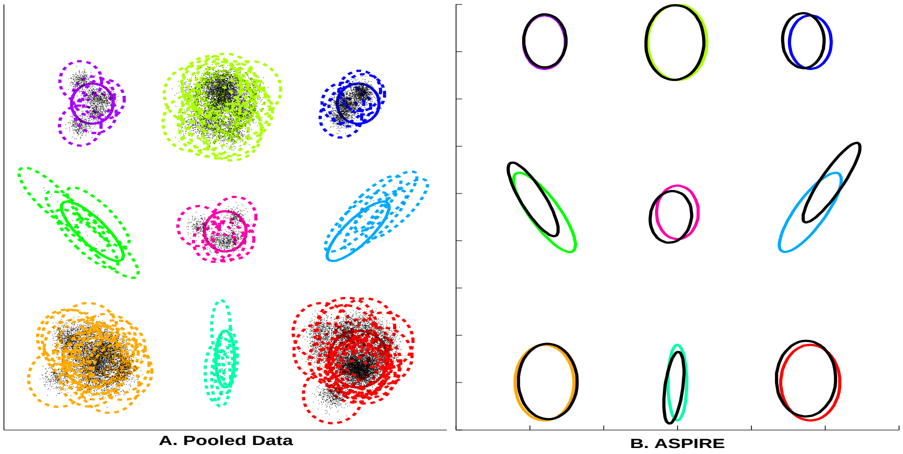

Figure 6: Pooled data and recovered distributions when $\kappa_1 = 0.2$.
