# Supplementary material for: A non-parametric Bayesian model for joint cell clustering and cluster matching: identification of anomalous sample phenotypes with random effects
Source: BMC Bioinformatics. 2014 Sep 24;15(1):314. doi: 10.1186/1471-2105-15-314 (PMC4262223; doi:10.1186/1471-2105-15-314)
Supplement: Supplementary file 3 — Additional file 3: Model Settings used in Experiments with PHS and AML Data Sets. (PDF 105 KB) [file 12859_2014_6631_MOESM3_ESM.pdf]

# Additional File 3

Model Settings used in Experiments with PHS and AML Data Sets

**June 23, 2014**

# 1 Model Settings used in Experiments with PHS and AML Data Sets

Parameter settings used for ASPIRE, DPGMM, FLAME, FlowPeaks, and HDPGMM in experiments with PHS and AML data sets are shown in Table 1 below.

Table 1: Model Settings used in Experiments with PHS and AML Data Sets.

| | |
|---|---|
| ASPIRE | m=d+2, $\kappa_0$=0.05, s=150log(d+1)/d<br>$\alpha = 1$, $\gamma = 1$<br>$\kappa_i \in \{0.1,\ 0.25,\ 0.5,\ 1\}$ (actual value selected to maximize Gibbs likelihood)<br>$\mu_0$=mean of all data points<br>$\Sigma_0$=I/s<br>burn-in sweeps = 750<br>post burn-in sweeps = 250 |
| DPGMM | m=d+2, $\kappa_0$=0.05, s=150log(d+1)/d<br>$\alpha = 1$<br>$\mu_0$=mean of all data points<br>$\Sigma_0$=I/s<br>burn-in sweeps = 750<br>post burn-in sweeps = 250 |
| FLAME | minimal number of clusters to be fit = 3<br>maximal number of clusters to be fit = 8<br>density = skew t<br>estimate mode = no<br>method for choosing optimal number of clusters = scale-free weighted ratio |
| flowPeaks | tolerance for merging clusters = 0.1<br>multiplier of the variance matrix S0 = 1<br>multiplier of the variance matrix S = 1.5 |
| HDPGMM | truncation level = 128<br>burn-in sweeps = 10,000<br>post burn-in sweeps = 100 |
