# Supplementary material for: A non-parametric Bayesian model for joint cell clustering and cluster matching: identification of anomalous sample phenotypes with random effects
Source: BMC Bioinformatics. 2014 Sep 24;15(1):314. doi: 10.1186/1471-2105-15-314 (PMC4262223; doi:10.1186/1471-2105-15-314)
Supplement: Supplementary file 4 — Additional file 4: Trace Plots of Cluster Proportions in Experiments with PHS and AML Data Sets. (PDF 467 KB) [file 12859_2014_6631_MOESM4_ESM.pdf]

Additional File 4

Trace Plots of Cluster Proportions in Experiments with PHS and AML Data Sets

June 23, 2014

1 Trace Plots of Cluster Proportions in Experiments with PHS and AML Data Sets

The trace plots of clusters proportions produced by ASPIRE on the AML and PHS data sets are shown in Figs. 1 and 2, respectively.

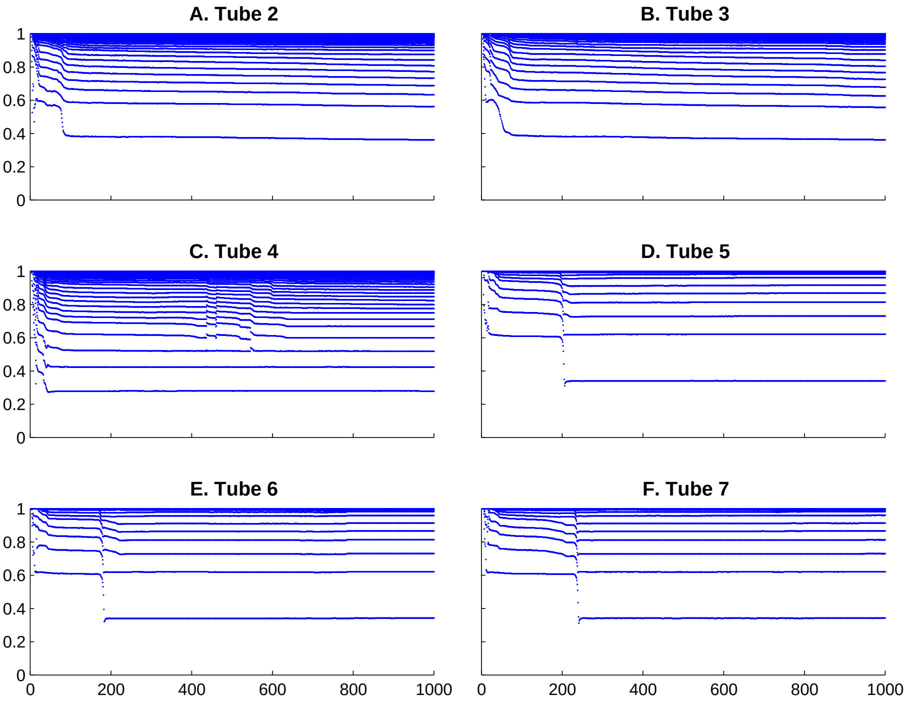

Figure 1: Trace plots of clusters proportions produced by ASPIRE on data from each of the six tubes in the AML data set.

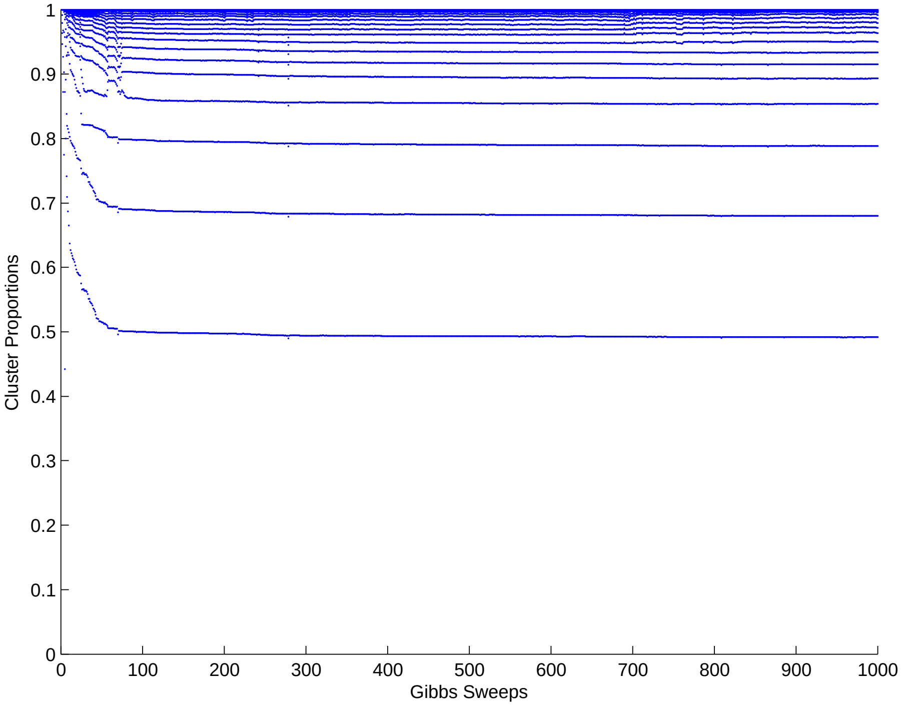

Figure 2: Trace plots of clusters proportions produced by ASPIRE on PHS data set.
